# The Use of Broadly Neutralizing Antibodies (bNAbs) in HIV-1 Treatment and Prevention

**DOI:** 10.3390/v16060911

**Published:** 2024-06-04

**Authors:** Jannifer Jasmin Thavarajah, Bo Langhoff Hønge, Christian Morberg Wejse

**Affiliations:** 1 Faculty of Health, Aarhus University, 8000 Aarhus C, Denmark; 2 Clinical Medicine, Department of Infectious Diseases, Aarhus University Hospital, 8200 Aarhus N, Denmark; bohonge@clin.au.dk (B.L.H.); wejse@clin.au.dk (C.M.W.); 3 GloHAU, Center of Global Health, Department of Public Health, Aarhus University, 8000 Aarhus C, Denmark

**Keywords:** HIV-1, broadly neutralizing antibodies, clinical trial, treatment, prevention, remission, cure strategy

## Abstract

Background: Although antiretroviral therapy (ART) effectively halts disease progression in HIV infection, the complete eradication of the virus remains elusive. Additionally, challenges such as long-term ART toxicity, drug resistance, and the demanding regimen of daily and lifelong adherence required by ART highlight the imperative need for alternative therapeutic and preventative approaches. In recent years, broadly neutralizing antibodies (bNAbs) have emerged as promising candidates, offering potential for therapeutic, preventative, and possibly curative interventions against HIV infection. Objective: This review aims to provide a comprehensive overview of the current state of knowledge regarding the passive immunization of bNAbs in HIV-1-infected individuals. Main findings: Recent findings from clinical trials have highlighted the potential of bNAbs in the treatment, prevention, and quest for an HIV-1 cure. While monotherapy with a single bNAb is insufficient in maintaining viral suppression and preventing viral escape, ultimately leading to viral rebound, combination therapy with potent, non-overlapping epitope-targeting bNAbs have demonstrated prolonged viral suppression and delayed time to rebound by effectively restricting the emergence of escape mutations, albeit largely in individuals with bNAb-sensitive strains. Additionally, passive immunization with bNAb has provided a “proof of concept” for antibody-mediated prevention against HIV-1 acquisition, although complete prevention has not been obtained. Therefore, further research on the use of bNAbs in HIV-1 treatment and prevention remains imperative.

## 1. What Are the Main Findings?

- Passive immunization with a single bNAb offer a transient reduction in HIV-1 viremia–short lived but significant.
- Combination therapy of ≥2 bNAbs maintain viral suppression for a longer period of time and delay time to rebound after analytical treatment interruption (ATI) in individuals with antibody-sensitive strains.
- Combination therapy enhances the HIV-1 specific T-cell response and changes both the size and composition of the intact proviral reservoir.
- Attaining prolonged viral suppression without treatment intervention, whether through ART or bNAb-based immunization, proves challenging due to reservoir diversity and pre-existing or de novo escape mutations. Only a very small subset of individuals has acquired post-treatment viremic control.
- The main barriers of the clinical implementation of bNAbs as an alternative treatment to ART are the latent reservoir, viral resistance (pre-existing and de novo mutations), and elimination half-life of bNAbs.

## 2. Introduction

Often referred to as ‘the plague of the 21st century’, HIV-1 remains a major public health issue that continues to claim thousands of lives every year despite effective antiviral treatment and tools of prevention [1]. Since its emergence in the early 1980s, researchers have been working towards a cure that eradicates the virus completely, yet a sterilizing cure remains elusive. While antiretroviral therapy (ART) has managed to effectively suppress viral load to undetectable levels, allowing it to be untransmittable when taken as prescribed, several challenges persist [2]. Factors such as the burden of daily and lifelong adherence to ART, long-term toxicity with ART, increasing drug-resistant strains, non-AIDS-related comorbidities, accessibility, social stigma, and the persistent latent reservoir further underlines the need for an alternative and longer-acting therapeutic approach aimed at persistent remission or potentially complete eradication [3,4,5,6]. At the very least, achieving ART-free remission without the need of daily and lifelong ART is imperative. An approach that recently has garnered considerable attention is immunization with broadly neutralizing antibodies, bNAbs. Preclinical studies conducted in non-human primates have demonstrated the significant suppression of viremia and delay in viral rebound [7,8,9]. With bNAbs exhibiting longer elimination half-lives (T_1/2_) compared to currently available ART, passive immunization with bNAbs could emerge as a superior treatment option, particularly advantageous for individuals facing challenges relating to compliance and accessibility to ART.

### 2.1. The Difficulties with HIV-1 Treatment, Prevention, and Cure

Achieving ART-free remission and potentially a cure for HIV-1 presents numerous challenges primarily due to the inherent nature of the virus and the limitations of current ART. (1) First, the main barrier to achieving a sterilizing cure is the latent proviral reservoir of HIV-1, predominantly residing in HIV-infected CD4+ T-cells [10]. Due to the establishment of a proviral reservoir, HIV-1 is able to lay dormant and effectively evade recognition by the immune system. Consequently, the reservoir allows the virus to remain undetected and re-emerge upon ART cessation, eventually leading to viral rebound. (2) Second, HIV-1 is one of the most genetically diverse retroviruses, characterized by its high mutation rate, genetic recombination, rapid viral replication, and the error-prone nature of its reverse transcriptase enzyme (RT) [11]. These attributes contribute to the presence of heterogenous strains of the virus to exist within each individual living with an HIV-1 infection and drive the emergence of ART-resistant mutations [11,12]. These drug-resistant strains are on the rise in some places in the world and could render treatment regimens of ART ineffective in the future [13,14]. (3) Third, considering that only the envelope spike protein (Env) is susceptible to neutralization, coupled with the fact that Env is sparsely expressed on the surface of HIV-1 [15], it becomes imperative for antibodies to exhibit sufficient breadth and potency to effectively target and neutralize the diverse strains. Additionally, the density of Env expressed on reactivated HIV-1-infected cells remains low, further emphasizing the need for highly effective antibodies in antibody-mediated immunotherapy [16]. (4) Fourth, ART successfully suppresses viral replication and prevents disease progression; however, it is unable to reach and eradicate the proviral reservoir or boost host antiviral immunity [17]. Consequently, because the proviral reservoir remains unaffected by ART, strict daily and lifelong adherence is necessary to prevent viral rebound. (5) Moreover, while it is commonly accepted that drug-resistant mutations (DRMs) do not emerge in virally suppressed individuals during ART, studies have indicated that HIV-infected ART-naïve individuals are a major source of DRMs [18]. The prevalence of transmitted drug-resistance (TDR) was found to be at a moderate level among ART-naïve patients with acute HIV-1 infection in a meta-analysis from 2021. Interestingly, the prevalence was higher in higher-income countries compared to low-income countries [19]. Additionally, inadequate adherence to antiretroviral treatment for HIV-1 can led to increased drug resistance [20]. So, when ART-treated individuals fail to adhere to treatment as prescribed, ongoing viral replication can foster and drive the development of escape mutations. (6) Finally, sustained long-term adherence to ART, life-long in this case, poses significant challenges for many individuals, irrespective of available resources. Several factors contribute to these difficulties, including socioeconomic conditions, government policies, healthcare infrastructure, the social stigma associated with HIV, and lack of access to healthcare services. Yet, on the other hand, accessibility does present an additional hurdle to some extent. According to the UNAIDS 2022 report, around 76% of all people living with HIV (PLWH) had access to ART (ranging 50–83%); this means that 9.2 million PLWH did not have access to ART in 2022 [1].

### 2.2. Broadly Neutralizing Antibodies (bNAbs)

A solution that addresses the above-mentioned challenges are bNAbs. Developing antibodies with sufficient breadth to target the diverse strains of HIV-1 viruses poses a significant challenge in the fight against HIV-1. Nonetheless, approximately 10–30% of individuals chronically infected with HIV-1 develop antibodies exhibiting exceptional breadth, capable of neutralizing a wide array of the circulating viral strains [21]. These antibodies, termed ‘broadly neutralizing antibodies’ (bNAbs), target conserved regions on the trimeric envelope glycoprotein found on the surface of HIV-1. The Env proteins are the only viral protein expressed on the surface of HIV-1 and are covered by a highly dense glycan shield [17,22,23], rapidly shifting glycans that shield potential target sites from neutralization by antibodies but not receptor binding. In other words, the glycan shield facilitates the evasion of the immune system [24]. Additionally, the envelope spike protein is highly genetically diverse and only expressed at a relatively low density on the surface of the virions [16,22]. In 1994, the first bNAb, b12, targeting the CD4-binding site of the Env protein, was isolated from an asymptomatic HIV-1-infected individual [25]. Subsequent advancements have resulted in the isolation and characterization of different classes of bNAbs that are up to 100-fold more potent [26].

**Figure 1:**
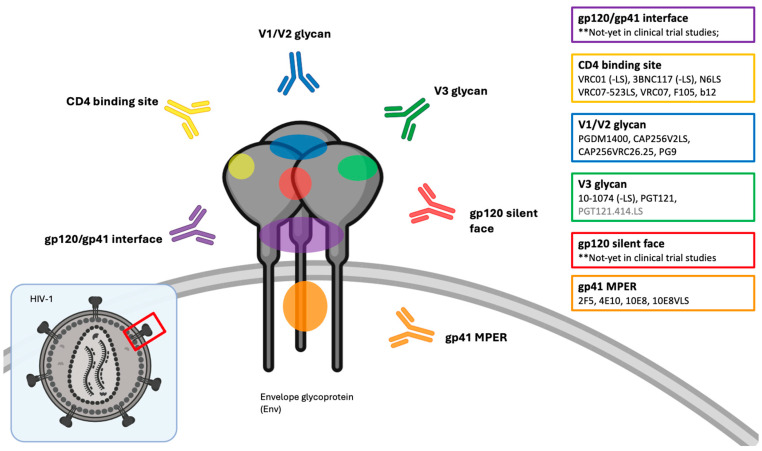
Targets of anti-HIV-1 broadly neutralizing antibodies (bNAbs) on the envelope spike protein (Env), including first- and second-generation bNAbs, that are used in vivo clinical trial studies. The figure is created using BioRender.com (accessed on the 29 April 2024).

There are several conserved regions on the HIV-1-Env trimer that are particularly susceptible to neutralization, to which these bNAbs bind. These include the CD4-binding site, V3-glycan, V1/V2 glycan, gp41 membrane-proximal external region (MPER), gp120-gp41 interface, and the gp120 silent-face center [25,27,28] (Figure 1).

### 2.3. Mechanism of Action

**Figure 2:**
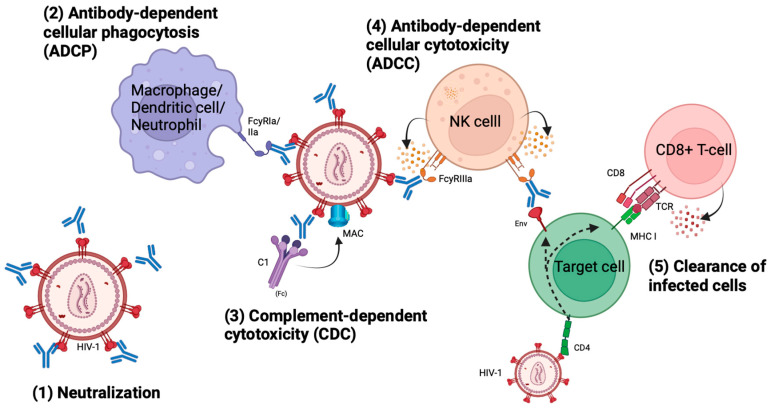
Effector functions of broadly neutralizing antibodies. Through their Fc-domain, bNAbs are able to elicit antibody-dependent cellular phagocytosis (ADCP), antibody-dependent cellular cytotoxicity (ADCC), complement-dependent cytotoxicity (CDC), and clearance of infected cells expressing Env on their surfaces. The figure is created using BioRender.com (accessed on the 29 April 2024).

The main characteristic feature of antibodies lies in their ability to neutralize pathogens through their variable region, the Fv domain. The binding of the Fv domain effectively impedes the attachment of HIV-1 to CD4+ cells, thus inhibiting viral entry and subsequent infection of host cells (Figure 2). Additionally, antibodies exert antiviral effects beyond neutralization through the constant region, the Fc domain, which enables phagocytosis and effector-cell-mediated killing by opsonization. By tagging free virions or infected cells expressing Env, antibodies facilitate recognition by effector cells, subsequently initiating antibody-dependent cellular phagocytosis (ADCP), antibody-dependent cellular cytotoxicity (ADCC), and compliment-dependent cytotoxicity (CDC) (Figure 2) [29]. This opsonization process enables bNAbs to aid in the clearance of the viral reservoir of HIV-1 [30].

Broadly neutralizing antibodies offer several unique characteristics in their mechanism of action compared to neutralizing antibodies that make them highly effective in targeting and neutralizing HIV-1. Firstly, as previously mentioned, bNAbs have exceptional ability to recognize and target the conserved regions on the Env glycoprotein [31]. These conserved epitopes are less variable among the various strains of HIV-1, allowing bNAbs to target and neutralize a broader range of diverse HIV-1 strains (increased breadth). However, differences in epitope availability and the conformation of Env may compromise the efficacy of bNAbs in targeting these cells for effector-cell-mediated killing [15,32]. Moreover, bNAbs are uniquely capable of penetrating the glycan shield surrounding the surface of HIV-1, enabling them to better bind the conserved epitopes on Env [33].

Consequently, bNAbs possess both therapeutic and prophylactic potential against HIV-1 infection, given their ability to mediate the killing of free circulating virions as well as Env-expressing infected host cells, i.e., maintain viral control and eliminate the latent reservoir. Unlike antiretroviral treatment, bNAbs have the capacity to elicit both antiviral activity and immunological effects through their Fc domain (Figure 2), underscoring the potential of bNAbs to contribute to the fight against HIV-1. In individuals with an advanced HIV-1 infection, various mechanisms are responsible for an impaired anti-HIV-1-specific response (including CD4+ and CD8+ T-cells) causing the immune system to get exhausted the longer the infection persists [34,35]. Thus, the passive infusion of bNAbs may greatly help the otherwise exhausted immune system.

Previous studies on passive immunization with monoclonal antibodies (mAbs) have demonstrated great efficacy in various context, including the prevention of hepatitis B, measles, and respiratory syncytial virus (RSV) in high-risk infants [36,37]. Moreover, immunization with mAbs have proven highly effective in the treatment of several cancers and autoimmune diseases [38,39]. Consequently, antibody-mediated immunization could be a valuable tool in HIV-1 treatment and prevention, serving as either an adjuvant or an alternative to ART. Hence, the purpose of this review is to assess the current status of passive immunization with broadly neutralizing antibodies from clinical trial studies in humans (in vivo studies), focusing on their therapeutic and prophylactic potential.

## 3. Findings from Clinical Trial Studies

The antibody-mediated treatment of HIV-1 has significantly advanced in recent years from pre-clinical studies to clinical trials in vivo. Recent data from clinical trial studies have yielded renewed excitement for immunological approaches for HIV-1 treatment and prevention, particularly, passive immunization with bNAbs, as they hold great therapeutic and prophylactic potential. Here, we present the main outcomes from the clinical trial studies on the passive immunization of bNAb(s) in various clinical trial phases.

### 3.1. First Generation bNAbs

The earliest discovered bNAbs, characterized for their ability to neutralize a diverse array of HIV-1 strains, are referred to as “first-generation bNAbs”. Passive immunization trials involving these first-generation bNAbs, namely b12, 2G12, 4E10, 2F5, and F105, have been evaluated in preclinical trials conducted in non-human primates, demonstrating reduction in viremia and even complete protection against simian–human immunodeficiency virus infection (SHIV) in macaques [40,41,42,43]. In human clinical trials, the passive infusion of these first-generation bNAbs were generally safe and well tolerated (an overview of these trials is depicted in Table 1) [44,45,46,47]. However, passive immunization with 2F5, 2G12, and 4E10, administered to ART-naïve HIV-1-infected individuals resulted in only a transient reduction in the viral load [44]. The rapid emergence of viral escape variants limited the duration of viral load suppression, resulting in viral rebound. Notably, two studies involving ART-treated HIV-1-infected individuals, reported a significant delay in time to rebound following analytical treatment interruption (ATI) when passively immunized with 4E10, 2F5, and 2G12 up to 24 weeks [48,49]. However, the loss of viremic control was strongly associated with the emergence of resistant strains to 2G12, with subsequent analysis confirming this [48]. Nevertheless, first-generation bNAbs did not clear the infections nor maintain viral suppression, mainly due to the rapid emergence of escape variants [48]. Escape mutations were frequently detected, ultimately leading to viral rebound [50]. Moreover, these effects were only observed in a small subset of study participants. Lastly, 2F5 and 4E10 have been found to exhibit autoreactivity [51,52].

Thus, despite the initial excitement surrounding the therapeutic and prophylactic potentials of first-generation bNAbs observed in in vitro and non-human primates, their unfavorable characteristics and limited antiviral activity led researchers to desert the idea of passive antibody-mediated immunotherapy. However, the studies provided proof-of-concept for passive immunization with bNAbs in humans, suggesting that bNAbs with increased breadth and potency is imperative for future studies.

### 3.2. Second-Generation bNAbs

As technological advancements in isolation techniques of antibodies progressed, leading to improved understanding of their mechanisms and structures, a new generation of bNAbs with increased breadth and potency were discovered, subsequently termed second-generation bNAbs [54]. Clinical trial studies of these bNAbs administered intravenously (IV), subcutaneously (SC), and intramuscularly (IM) have consistently demonstrated safety and tolerability [55,56,57,58,59,60,61,62,63,64,65,66,67,68,69,70]. The majority of adverse effects observed were associated with the method of administration and were typically mild (grade 1, 2, or maybe 3), such as myalgia [63,65,66], and resolved within a few days [57,63]. These observations hold true for both single and multiple infusions administered to both infected and non-infected/healthy individuals. Additionally, no anti-drug antibodies (ADAs) against the bNAbs were detected [47,57,60,65,70], which is noteworthy as it could have impacted antiviral efficacy and accelerated bNAb decay. The only exception was the presence of anti-3BNC117 and anti-10-1074 antibodies found in the study by Cohen et al. in healthy HIV-1-negative individuals; 4 out of the 15 participants developed specific anti-bNAb antibody responses against one of the bNAbs. Moreover, the anti-bNAb responses that were elicited did not impact elimination half-life nor result in any adverse events [71]. These favorable safety profiles of bNAbs can likely be attributed to their high specificity and affinity for targets, consequently reducing the likelihood of eliciting unexpected adverse effects.

### 3.3. Monotherapy with a Single bNAb

Early clinical trials involving second-generation broadly neutralizing antibodies, including N6LS, VRC01, 3BNC117, 10–1074, and PGT121, are outlined in Table 2.

Passive immunization with a single bNAb provided a rapid but transient and moderate reduction in viral load in both viremic and aviremic HIV-1-infected individuals [58,59,64,66,69,70,76]. Although significant, this reduction in viremia was often short lived as viral rebound rapidly occurred in most individuals even with bNAb-sensitive strains [58,59,66,67]. In the study by Schoofs et al., HIV-1 from peripheral blood mononuclear cells (PBMC) of nine viremic HIV-1 infected individuals were cultured and tested at day 0 and at week 4. On day 0 all but one of the cultured viruses were sensitive to 3BNC117, while increasing resistance was observed in most individuals at week 4, indicating selection for escape variants [75]. Nonetheless, a select few ART-treated individuals with initial low viral loads maintained viral suppression for multiple weeks [62,66,67,69,70,76]. However, no effect on viremia was observed in chronically HIV-1-infected participants in some cases [61,62]. Additionally, some individuals did not respond to the bNAb at all due to pre-existing bNAb-resistant HIV-1 strains [66].

The importance of HIV-1 strain sensitivity to bNAbs has been demonstrated in several studies. Passive immunization with 3BNC117 maintained viral suppression for up to 28 days in ART-naïve but aviremic individuals with a reduction of up to 2.5 log_10_ copies/mL (range: 0.8–2.5 log_10_ copies/mL) [66]. Up to 19 weeks was observed in ART-treated HIV-1 infected participants with 3BNC117-sensitive strains undergoing ATI [67]. As the median time to rebound for historical controls is around 2.6 weeks [67], the enhanced antiviral efficacy of 3BNC117 in both ART-treated and -naïve participants is evident. Additionally, 3BNC117 was found to enhance host humoral responses to HIV-1 in ART-naïve viremic HIV-1-infected individuals [75]. However, and most importantly, monotherapy with 3BNC117 alone was insufficient in sustained viral control due to escape mutations; rebound was found to arise predominantly from a single provirus [67]. The antiviral effects of VRC01 were, on the other hand, more limited; modest delay in viral rebound was observed in aviremic (ART-treated) chronically infected individuals [59,73]. Despite this modest delay in viral rebound during ATI, all participants eventually rebounded [73]. Additionally, participants who showed faster decay of VRC01 in serum consequently rebounded more rapidly, while participants with VRC01-sensitive strains rebounded later. Interestingly, viremia rebounded despite the absence of HIV-1 adaptation to VRC01. The effect of a single VRC01 on HIV-1 reservoir size and composition was likewise limited and only demonstrated in two studies; Lynch et al. [58] and Riddler et al. [61] both found that VRC01 alone did not reduce cell-associated viral loads (HIV-DNA) in ART-treated chronically infected individuals. Similarly, passive immunization with 3BNC117 did not measurably reduce the size of the latent reservoir in virologically suppressed HIV-1-infected individuals [68].

In summary, while monotherapy with a single bNAb led to initial viral suppression, it proved inadequate in maintaining durable viral suppression due to the emergence of HIV-1-resistant variants. Viral rebound occurred in the majority of the study participants due to either existing, de novo resistance mutations or bNAb-titer decay, thereby enabling viral escape and the rebound of viremia. Analogously to ART, the use of a combination therapy comprising at least two bNAbs targeting non-overlapping epitopes appears warranted and may be essential to mitigate viral escape.

### 3.4. Combination Therapy with ≥2 bNAbs

A combination of two or more bNAbs provides durable suppression of viral load in both viremic [77,78] and virologically suppressed [79,80,81] HIV-1-infected individuals. In general, a combination therapy of multiple bNAbs was safe and well-tolerated and did not result in any serious adverse event related to the passive immunization of the treatment [71,78,82,83,84]. One exception is the MPER-targeting bNAb, 10E8VLS, which was assessed alone and in combination with VRC07-523LS via subcutaneous injections in healthy adults [85]. Due to moderate and severe local reactogenicity (Grade 2 and Grade 3), this study was voluntarily terminated. The subcutaneous injection resulted in moderate reactogenicity in 5/8 healthy non-HIV-1-infected individuals, while one participant experienced severe local reactogenicity. Additionally, the elimination half-life was shorter than expected (8.1 days) [85]. An overview of the clinical trials evaluating the combination of multiple bNAbs is depicted in Table 3.

While monotherapy with a single bNAb showed limited efficacy in maintaining long-term viral suppression in most participants due to the emergence of HIV-1-resistant variants, combination therapy with at least two bNAbs are able to provide a longer-lasting viral suppression in a majority of HIV-1-infected individuals by restricting viral escape. Moreover, combining different classes of bNAbs provided enhanced neutralization efficacy, suggesting an additive effect in controlling viral replication. The combination of 3BNC117 and 10-1074 is more effective in suppressing viremia than either antibody alone [77]. When administered to aviremic HIV-1-infected individuals undergoing ATI and with bNAb sensitivity, significant delay in viral rebound was observed for up to 21 weeks. Additionally, none of these individuals developed resistance to any of the bNAbs [79]. On top of that, in viremic, untreated participants the same combination of bNAbs resulted in significant delay as well for up to 3 months in individuals with antibody-sensitive strains [77]. Therefore, the combination of 3BNC117 and 10-1074 offers significant viral suppression and delay in viral rebound in both viremic and virally suppressed individuals, given that they are sensitive to both bNAbs. Remarkably, a significant delay in viral rebound, lasting up to a year, was observed in two individuals following ART cessation when treated with a combination of 3BNC117 and 10-1074 [80]. Although the study involved a small sample, this observed delay is the longest reported to date. Several other studies have documented multiple instances of prolonged viral suppression and delayed viral rebound [77,79,80,81,86]. For instance, in viremic HIV-1-positive individuals undergoing ATI, sustained viral suppression for up to 43 weeks was observed, provided no antibody-resistant strains were present at baseline and that the individuals had initiated ART during the acute/early phase of HIV-1 infection [81], likely causing a lower degree of diversification of viral and proviral strains plus explained by the prescreening of all participants for antibody sensitivity [77,79,81]. In another study by Mendoza et al., the suppression of the viral load was obtained in nine HIV-1-infected individuals with antibody-sensitive viral reservoirs during ATI for between 15 and more than 30 weeks, when administered a combination of 3BNC117 and 10-1074. Additionally, none of the participants developed resistant viruses to either of the bNAbs [79]. Therefore, to optimize bNAb-mediated treatment of HIV-1, important factors to consider include viral diversification, pre-existing antibody sensitivity, and sufficient breadth of the bNAbs to prevent escape mutations. The combination of 3BNC117 and 10-1074 also resulted in a reduction in intact proviral reservoir but no measurable change in defective proviral reservoir [80], thereby providing evidence of bNAbs’ antiviral effect on HIV-1 infected host cells. Additionally, this combination also provided significant increase in HIV-1 Gag-specific CD8+ and CD4+ T-cell responses in HIV-1 infected individuals during ATI [86], demonstrating that bNAbs-mediated therapy is associated with enhanced HIV-1-specific T-cell responses. On the contrary, however, a study by Sneller et al. did not observe any statistical significant change in the frequency of CD8+ T-cells, levels of cell-associated HIV RNA or intact proviral DNA in HIV-1-infected individuals whose plasma viraemia remained suppressed >30 weeks after ATI, suggesting that 3BNC117 and 10-1074 does not have a significant effect on the persistent HIV-1 reservoir [81]. From studies on elite non-progressors, it becomes evident that a strong and highly functional HIV-specific CD8+ T-cell response is associated with spontaneous viral control [87]. Therefore, further studies are needed to determine the impact of bNAbs on CD8+ and CD4+ T-cell responses and to understand how these effects influence the size and composition of HIV-1 reservoir.

Overall, these studies demonstrates that combinations of bNAbs are generally safe (with few exceptions) and suggest that the combination therapy is able to maintain viral suppression for a longer time compared to both monotherapy and currently available ART (when taken as prescribed) during ATI, given that the pre-existing viruses are bNAb-sensitive and adequate elimination half-lives to maintain therapeutic levels are obtained. Despite this, viral escape is still a pressing issue using dual bNAb combination therapy. Out of 11 virally suppressed HIV-1-infected individuals with no detectable resistant viruses in the pre-infusion latent reservoir, 7 participants rebounded during ATI after receiving 3BNC117 and 10-1074 [79]. Interestingly, the study by Mendoza et al. found that viral rebound never occurred when the concentration of both administered bNAbs remained above 10 μg/mL, highlighting the importance of bNAb titers in serum and their corresponding half-lives. Consequently, due to viral escape observed with dual bNAb therapy, the exploration of triple-bNAb therapy has been undertaken. These triple-bNAbs combinations, which includes modificed bNAbs with the LS-mutations, have been evaluated in clinical trials to ensure a stronger suppression of viremia and inhibit viral escape [78,82]. A single infusion of PGDM1400, PGT121, and VRC07-523LS in viremic ART-naïve individuals rapidly reduced viral load but only transiently as viral rebound occurred just 20 days after bNAb infusion in all participants. Viral rebound was associated with pre-existing partial or complete resistance against PGDM1400 and PGT121 in vitro and bNAb-titer decay [78]. Therefore, for optimal bNAb-mediated treatment the following must be considered; combination of what bNAbs, pre-screening for antibody-sensitivity, stage of infection (acutely or chronically infected), bNAb half-life in vivo (to ensure high serum concentration), and if the participants are ART-treated or -naïve. Currently, an on-going study from 2021 (NCT04319367), known as the “RIO” study, is evaluating the effects of longer-acting bNAbs, 3BNC117-LS, and 10-1074-LS in participants initiating ART early after HIV-1 acquisition during primary HIV-1 infection and with no evidence of viral insensitivity to either bNAbs.

Numerous on-going studies in early clinical phases are currently evaluating longer-acting and more potent bNAbs [88,89]. For example, the phase 2 clinical trial, RHIVIERA-02, that aims to access the combined effects of antiretroviral therapy (ART) with long-acting bNAbs in newly diagnosed individuals during early-stage infection [89]. The outcomes of these trials will be essential to better discern the potential role that bNAbs could play in HIV-1 treatment.

### 3.5. Combination Therapy with bNAb(s) and Immunostimulatory Agents

In pursuit of a potential cure of HIV-1, the eradication of the latent reservoir is pivotal. Geabler et al.’s study demonstrated a reduction in the size of intact proviral reservoir following infusions of 3BNC117, 10-1074, and ART [80]. However, contrasting results were observed in other studies involving VRC01, where no measurable impact on the latent reservoir could be demonstrated [58,61]. To address this challenge, latency-reversing agents (LRAs) have been explored in conjunction with bNAbs to induce proviral activation and subsequently target the latent reservoir of HIV-1 (so-called “shock and kill” approach). Data from studies in SHIV infected rhesus monkeys highlight the potential of combining a latency-reversing agent (LRA), such as toll-like receptor (TLR) agonists, and bNAbs to target and eliminate the viral reservoir for [90,91,92]. For instance, in the studies by Borducchi et al. [90] and Barouch et al. [91], viral rebound was only observed in 6 out of 11 and 10 out of 17 monkeys that received a combination of TLR7-agonist and PGT121, respectively. However, recent studies have not shown the same degree of efficacy in reducing viremia and viral reservoir in humans (see Table 4) [93,94]. In a study by Gruell et al., which assessed the combination of 3BNC117 and romidepsin, a histone deacetylase inhibitor, no significant change in HIV-1 DNA (reservoir), immune responses, or delay in viral rebound were observed when compared to romidepsin alone. Additionally, although deemed generally safe, two severe adverse events were observed during follow-up [93]. Conversely, in a similar study by Gunst et al. assessing 3BNC117 and romidepsin, a significant reduction in the frequency of CD4+ T-cells containing intact proviruses was observed in addition to accelerated clearance of infected cells and enhanced HIV-1-specific CD8+ T-cell responses [95]. Although both trials evaluated the same agents, they yielded disparate outcomes. This discrepancy could potentially be attributed to the fact that Gunst et al. enrolled newly diagnosed participants, resulting in the decreased diversification of viral strains, and immediately initiated ART to keep viral replication suppressed, thereby enabling the bNAbs to interact more effectively with the remaining viruses [95]. By contrast, Gruell et al. evaluated chronically HIV-1-infected individuals [93]. These findings suggest a possible window of optimal use for bNAb-mediated interventions in HIV-1, further clinical research is, therefore, warranted. Moreover, no added benefits of the TLR9-agonist, lefitolimod (LEFI), were observed when comparing the combination of LEFI with 3BNC117 and 10-1074 versus the 3BNC117 and 10-1074 alone on virological control [96].

### 3.6. Elimination Half-Time

The burden of daily and life-long oral medication in ART can negatively affect adherence, thus driving ART resistance and escape variants as previously mentioned. In order for bNAbs to replace ART and accommodate the burden of daily adherence, bNAbs would have to maintain viral suppression for longer with less frequent dosing. To decrease the dosage frequency of bNAb administration, modified bNAbs (-‘LS’) variants have been engineered to increase the epitope affinity and extend half-life (Table 5 presents the half-lives of both the LS- and their original versions of bNAb), thus enabling enhanced antiviral activity and immunological effects. These bNAbs have been engineered with two specific mutations, Met428Leu (M428L) and Asn434Ser (N434S), which enhance their binding affinity to the neonatal Fc receptor (FcRn) found on endothelial cells at a low pH. This increased affinity-limiting lysosomal degradation, promoted recycling, and therefore extended the serum half-life of the antibodies [98]. Moreover, the tissue distribution of FcRn allows bNAbs to be present at key mucosal compartments, potentially providing HIV-1 protection at the site of exposure and aiding in the prevention of HIV-1 acquisition [99]. In a study by Gaudinski et al., VRC01-LS, the modified version of VRC01, was administered to HIV-1-uninfected individuals and showed an elimination half-life of 71 days (±18) [63]. Contrarily, the elimination half-life of VRC01 in HIV-1-uninfected individuals was approximately 17 days (Table 5) [66]. The half-life of bNAbs appears to be shorter in HIV-1-infected individuals compared to uninfected individuals, likely due to the rapid clearance of bNAb-mediated complexes. This is further underlined in Julg et al.’s study: the half-life of PGDM1400 was approximately 20 days when given alone in HIV-1-negative individuals (Table 5). However, in HIV-1-positive individuals with viraemia, the half-life dropped to 11 days [78]. The use of bNAbs with -LS modification will likely be crucial to maintain serum titer levels associated with protection and especially beneficial in resource-poor settings. On the other hand, recently long-acting injectable ART (LA-ART) have been developed and studied in clinical trials, showing the high acceptability, tolerability, and levels of virological suppression, possibly prompting their use in the near future [100]. Additionally, while the LS mutations are generally designed to preserve, and not eliminate, the interaction with other Fc receptors involved in Fc-mediated effector functions such as ADCC and CDC, any modification to the Fc region has the potential to affect these interactions. Yet, a study conducted in non-human primates found that the binding to Fc*γ*RIIIa was retained, and functions including ADCC was maintained at similar levels to the wildtype bNAb [99]. Nonetheless, further research in humans is warranted. The preservation of the Fc-mediated effector functions in bNAbs is significant as they hold the potential to enhance existing immune responses and directly eliminate infected cells, consequently interfering with the viral reservoir of HIV-1. Currently, the selection of bNAbs in clinical trials are not considered based on their Fc effector functions. However, considering the potential benefits of including antibodies that mediate broad and potent Fc effector functions could be valuable for future research and development.

### 3.7. Post-Treatment Viral Control

While immunotherapy with two or more bNAbs was insufficient in preventing viral escape, a small number of HIV-1-positive participants were able to maintain sustained viral suppression following ATI with viral loads of less than 400 copies/mL. These post-treatment controllers (PTCs) suppress viral replication in the absence of ART, subsequently delaying viral rebound for up to years. While spontaneous HIV-1 controllers or “elite controllers” are able to naturally maintain low viral loads at undetectable levels, they do, however, only represent a small subset of HIV-1-infected individuals, approximately 0.5% [102]. On top of that, so-called “exceptional elite controllers” are defined as individuals who are able to suppress viremia and delay disease progression for more than 25 years in the absence of ART [103]. In HIV-1-positive participants receiving immunotherapy with bNAbs, PTCs have also been described, but only rarely. For instance, post-treatment viral control was observed in two individuals remained suppressed for over 30 weeks after ART discontinuation [79]. Although, maintaining undetectable viral loads post-ART treatment may be associated with early antiviral treatment initiation, further research is implicated to include those with a chronic infection and to understand the mechanisms enabling this post-treatment viraemic control. These cases with post-treatment controllers are particularly important as they offer insight into potential strategies for achieving HIV-1 remission.

### 3.8. HIV-1 Prevention

Currently, multiple tools of prevention exist against HIV-1, including behavioral (male circumcision and condoms) and biomedical pre-exposure prophylaxis (PrEP) [104,105]. Based on multiple randomized controlled studies, there is a negligible risk of sexual and mother-to-child transmission of HIV-1 when a seropositive partner/mother adheres to the daily regimen of ART (aka. PrEP) and, therefore, maintains viral loads under 200 copies/mL (undetectable = untransmittable) [106]. However, the efficacy of PrEP is not 100% and, like ART, require daily adherence, i.e., efficacy of PrEP is highly dependent on adherence [107,108]. On top of that, UNAIDS reported in 2019 that out of 69 countries implementing PrEP, 56 countries reported barriers that limited access to PrEP, cost and availability were among the main hurdles [109]. As such, alternative strategies and approaches for HIV-1 prevention remain a priority, especially for people at high risk.

In this regard, bNAbs have recently garnered attention in the pursuit of improving and diversifying the biomedical options for HIV-1 prevention. Preclinical trials of passive immunization with bNAbs have provided complete prevention from HIV-1 acquisition in non-human primates [8,110,111]. These studies have provided valuable insight into the prophylactic potential of bNAbs, although challenges remain in predicting their effectiveness in humans due to differences in viral diversity [112]. As of yet, no clinical trials in humans have successfully managed to recreate this outcome. While several bNAbs have been assessed in phase 1 clinical trials in both HIV-1-infected and -uninfected individuals, only VRC01 has advanced to phase 2. This advancement was marked by the notorious antibody-mediated prevention (“AMP”) study by Corey et al., which investigated the CD4-binding site targeting bNAb, VRC01, in preventing HIV-1 acquisition. The study found that VRC01 demonstrated a 75% efficacy in prevention infection from HIV-1 strains that were highly sensitive to VRC01. However, VRC01 did not prevent the overall HIV-1 acquisition better than placebo. The AMP-study comprised two trials: (1) the HVTN 704/HPTN 085 trial, which assessed at-risk cisgender men and transgender individuals from America and Europe, and (2) the HVTN 703/HPTN 081 trial, which involved at-risk women in sub-Saharan Africa. Participants in both trials were randomly assigned to receive either a high dose (30 mg/kg) or low dose (10 mg/kg) of VRC01 or placebo [74]. While the study provided a proof of concept for using bNAbs as a preventative tool regardless of gender and region-specific clade, it simultaneously highlighted the limitations of VRC01 in HIV-1 prevention. Specifically, less than 30% of circulating strains exhibited antibody sensitivity of <1 μg/mL IC_80_, thus highlighting the need for either optimized bNAbs (with increased potency and breadth) or a combination of multiple bNAbs targeting non-overlapping epitopes, as VRC01 alone was not universally effective against all strains.

In summary, passive immunization with bNAbs offers a promising novel approach for HIV-1 prevention given their ability to neutralize circulating virions and infected host cells. The AMP study provides a strong rationale for pursuing a bNAb-mediated vaccine yet highlights its challenges as well; either higher bNAb serum titers or more potent and broader bNAbs are required for the successful prevention of HIV-1 acquisition in non-infected individuals. Consequently, further research is imperative before clinical implementation can be realized.

## 4. Challenges, Considerations, and Improvements

Alternative immunotherapeutic interventions with less adverse effects are warranted especially in HIV-1-infected patients with severe allergic reactions to ART and adherence problems. While bNAbs show promising therapeutic, prophylactic, and potentially curative effects in in vivo clinical trials, several questions and considerations remain open. The clinical trial studies presented in Table 3 highlight several considerations for devising an optimal combination strategy of passive immunization with bNAbs. In order to impede viral escape, the combination of bNAbs must have sufficient breadth and potency to ensure the complete neutralization of all viral strains. In addition, as emphasized in the study by Sneller et al. [81], it is equally important that the individual have bNAb-sensitive strains to the administered bNAbs. Early intervention in acutely HIV-1-infected individuals may limit the degree of proviral diversification [81,113], thus enabling bNAbs to better control viremia and reduce the emergence of escape mutations. Importantly, it should be emphasized that individuals are more commonly diagnosed with HIV-1 during the chronic stage of infection rather than the acute phase. This is primarily because the acute phase of HIV-1 typically presents with non-specific symptoms that can be mistaken for common illnesses. A recent study by Crowell et al. assessed how ART initiation during acute or early HIV-1 infection (AEHI) affects the viral reservoir and host immune responses [114]. They reported that early initiation during AEHI reduced but did not eliminate the persistence of HIV-1-infected cells in blood (circulating viral reservoir), suggesting that the efficacy of bNAbs is dependent on both baseline viral load, stage of infection, and the extent of established viral reservoir. Moreover, the selected bNAbs should target non-overlapping epitopes on the Env. Combining different classes of non-overlapping epitope targeting bNAbs has provided a greater neutralizing activity, suggesting an additive effect. A recent study by Sobieszczyk et al. assessed the triple-bNAb combination of PDGM1400, PGT121, and VRC07-523LS, revealing higher neutralizing activity compared to dual combinations of either PGVM1400 and VRC07-523LS or PGT121 and VRC07-523LS [82]. While the optimal number of bNAbs required for the sustained viremic control and the inhibition of viral escape remains uncertain, it may be dependent on each HIV-1-infected individual, considering the degree of diversification and their antibody sensitivity. Pre-screening individuals for antibody sensitivity may be necessary to observe the desired outcomes of bNAbs in a clinical setting, as pre-screening generally leads to enhanced viral load suppression and prolonged time to rebound during ATI [77,79,81]. However, due to cost and scalability, this option might not be a feasible goal. Moreover, it is important to consider that the sample collected for sensitivity screening might not fully represent all strains present in an individual with HIV-1 infection, further underscoring the complexities associated with HIV-1 treatment.

On a practical level, cost-effectiveness and equitable accessibility are crucial components to consider in order to clinically implement the bNAb-mediated immunotherapy for HIV-1 treatment and prevention. At present, the passive immunization using bNAbs for HIV treatment and prevention is not cost effective, particularly in terms of production, distribution, and overall sustainability. Despite the higher costs associated with bNAbs, it still holds potential as a long-acting alternative to ART. bNAbs can be administered intermittently (e.g., monthly or every few months), which can facilitate treatment for individuals in remote areas and thus improve adherence. Additionally, while current producing and distribution costs of bNAbs are higher than those of ART, ongoing research and advancements in biotechnology may help reduce these costs over time. It is particularly important to make bNAbs readily available in low-income countries as they carry the brunt of the pandemic. The COVID-19 pandemic underscored the inequitable distribution of vaccines on a global plan, emphasizing the urgency of addressing such disparities [115,116]. In this regard, various strategies are being explored; these include using adeno-associated virus (AAV) vectors to allow the sustained gene-mediated expression of bNAbs for both treatment [117] and prevention [118], as well as bi- or trispecific antibodies. To sustain the suppression of viremia without requiring multiple infusions of the antibodies, it is essential to achieve a stable bNAb titer in serum. When administering bNAbs by passive infusions, the half-lives of the bNAbs limit the concentration in serum. To mitigate this, gene transfer methods of adeno-associated virus (AAV) vectors could be utilized. Pre-clinical studies in SHIV-infected macaques have demonstrated prolonged viral suppression using AAV-mediated transfer of 3BNC117, 10-1074, and 10E8. However, a recent phase 1 study did not demonstrate a significant change in viral load during a 1–3-year follow-up of HIV-1-infected individuals [117]. Another possibility is the bi- and trispecific bNAbs. These next-generation bNAbs have been developed to further broaden the neutralization coverage of antibodies (breadth) [119,120,121] and could possibly facilitate superior clinical outcomes compared to the parental antibodies [122]. However, an important point to keep in mind before implementing these artificially developed antibodies is the development of antidrug antibodies, ADA, which can impede the therapeutic effects by neutralizing the bNAbs themselves, i.e., reduce the efficacy of the bNAbs in vivo [17]. According to Vaisman-Mentesh et al., the repeated administration of monoclonal antibodies, mAbs, can be highly immunogenic and cause the development of ADAs [123]. Here, the clinical trials that had multiple infusions have demonstrated minimal to no development of anti-bNAb antibodies [57,63,70,71,78]. However, the degree of engineering involved with the bi- or trispecific antibodies differs from that of bNAbs and could potentially elicit a stronger immunological response. In another study by Casazza et al., the AAV8-mediated delivery of VRC01 resulted in the development of non-idiotypic ADAs directed against the Fab portion of VRC01 in three out of eight participants (37.5%), which appeared to decrease the production of VRC01 in two out these three participants [117]. A comprehensive overview of all the possible strategies to improve immunotherapy is reviewed in [28].

Last but not least, while immunotherapy with bNAbs in HIV-1-infected individuals have been and are being studied extensively, their in vivo efficacy in HIV-2-infected individuals remains largely unexplored. This discrepancy can be attributed to several factors. HIV-2 is distinct from HIV-1, not only in genetic makeup and pathogenesis, but also in global prevalence [124]. Compared to HIV-1, HIV-2 is relatively uncommon, particularly outside of West Africa. As a result, fewer studies have focused on developing specific therapeutics against HIV-2. While some bNAbs may demonstrate cross-reactivity against HIV-1 and HIV-2 in in vitro studies, their efficacy in vivo may differ. Moreover, it has been proposed that HIV-2 could serve as an important model for HIV-1 studies due to its less severe pathogenesis and increased tendency towards latency [125]. Therefore, HIV-2 could be a valuable tool for testing and developing new methods applicable for HIV-1 cure. Nonetheless, developing effective therapeutics for HIV-2 infection is essential for addressing the needs of affected populations and advancing efforts towards global HIV infection control.

## 5. Concluding Remarks

In the absence of an effective vaccine, exploiting the potential of broadly neutralizing antibodies in HIV-1 treatment and prevention emerges as a promising therapeutic strategy to alleviate the burden of the HIV-1 epidemic. BNAbs offer a longer-acting therapeutic alternative to ART although remission remains elusive. Nonetheless, for bNAbs to be a viable alternative to ART, considerable improvements in their breadth, potency, and elimination half-life are imperative to curtail viral escape and, ultimately, prevent viral rebound. In general, the passive immunization of broadly neutralizing antibodies is deemed safe and well-tolerated in in vivo clinical studies of both HIV-1-infected and -uninfected individuals. The clinical trial outcomes of passive immunization with a single bNAb demonstrates the inadequacy to prevent viral rebound due to the emergence of bNAb-resistant strains of HIV-1, suggesting a potential benefit of combination therapy. Combination therapy of bNAbs provides greater efficacy in maintaining viral suppression and delaying viral rebound, especially in those with bNAb-sensitive strains. Moreover, bnAbs are able to maintain viral suppression for a prolonged time compared to ART, enhance the body’s own immune responses (including CD8+ T-cell activity), and reduce the size of the intact proviral reservoir when administered in combination with other non-overlapping targeting bNAbs. Yet, pre-existing and de novo escape mutations (reservoir diversification) continue to pose challenges in bNAb-mediated cure strategies, especially in those individuals with a chronic infection due to the increased diversification of viral reservoir.

Overall, bNAbs offer a multifaceted and promising approach for both treatment and prevention of HIV-1, with ongoing research aimed at optimizing potency and breadth and delivery methods and ensuring scalability for global utilization.

## Figures and Tables

**Table 1:** Clinical trials evaluating first generation broadly neutralizing antibodies.

Clinical Trial Study	Year	bNAb	Study Objective	Study Population	Ref.
Cavacini et al.	1998	F105	Safety and pharmacokinetic profile	HIV-1-infected individuals, n = 4	[53]
Armbruster et al. *	2002	2F5 + 2G12	Safety and pharmacokinetic profile	Asymptomatic, ART-naive HIV-1-infected individuals, n = 7	[45]
Stiegler et al. *	2002	2F5 + 2G12	Antiviral effects	Asymptomatic, ART-naive HIV-1-infected individuals, n = 7	[44]
Armbruster et al.	2004	4E10/ 4E10 + 2F5 + 2G12	Safety and pharmacokinetic profile	Asymptomatic, ART-naive HIV-1-infected individuals, n = 8	[46]
Joos et al. **	2006	4E10 + 2F5 + 2G12	Safety and pharmacokinetic profile	Chronic (8) and acutely (6) HIV-1-infected individuals undergoing ATI, n = 14	[47]
Trkola et al. **	2005	4E10 + 2F5 + 2G12	Efficacy	Chronic (8) and acutely (6) HIV-1-infected individuals undergoing ATI, n = 14	[48]
Mehandru et al.	2007	4E10 + 2F5 + 2G12	Efficacy	Acutely HIV-1-infected individuals undergoing ATI, n = 10	[49]

ART, antiretroviral therapy; ATI, analytical treatment interruption. * and ** are both part of the same study, respectively.

**Table 2:** Clinical trials of monotherapy with a single broadly neutralizing antibody.

Clinical Trial Study	Year	bNAb	Study Objective	Study Population	Ref.
(Widge et al.) CROI 2020	2020	N6LS	Safety and pharmacokinetics	HIV-1-negative individuals, n = 23	[55]
NCT04871113	2023	N6LS	Safety, pharmacokinetics, and antiviral activity	ART-naïve HIV-1-infected individuals, n = 63	[56]
NCT01993706 (Ledgerwood et al.)	2015	VRC01	Safety and pharmacokinetics	HIV-1-negative individuals, n = 28	[57]
NCT01950325 (Lynch et al.)	2015	VRC01	Safety and antiviral activity incl. viral suppression and cell-associated reservoir	ART-treated (aviremic) and -naïve (viremic) chronically HIV-1-infected individuals, n = 27	[58]
NCT02463227 NCT02471326 (Bar et al.)	2016	VRC01	Safety, pharmacokinetics, and antiviral activity incl. viral suppression and time to rebound	Aviremic chronically HIV-1-infected individuals undergoing ATI, n = 24	[59]
NCT02165267 (Mayer et al.)	2017	VRC01	Safety and pharmacokinetics	At low-risk HIV-1-negative individuals, n = 88	[60]
NCT02411539 (Riddler et al.)	2018	VRC01	Safety, pharmacokinetics, and antiviral activity incl. viral suppression and cell-associated reservoir	ART-treated chronically HIV-1-infected individuals, n = 40	[61]
NCT02664415 (Crowell et al.)	2019	VRC01	Safety and antiviral activity	Acutely ART-treated and virally suppressed HIV-1-infected individuals undergoing ATI, n = 23	[62]
NCT02797171	2019	VRC01	Safety, pharmacokinetics, and antiviral activity	HIV-1-negative individuals n = 80	[72]
(Cale et al.)	2020	VRC01	Antiviral activity incl. viral rebound	ART-treated, durably suppressed HIV-1-infected individuals undergoing ATI, n = 18	[73]
NCT02716675 NCT02568215 (Corey et al.)	2021	VRC01	Prevention efficacy	At-risk HIV-1-negative cis-gender men, transgender individuals (n = 2699) and women from sub-Saharan Africa (n = 1924)	[74]
NCT02599896 (Gaudinski et al.)	2018	VRC01-LS	Safety and pharmacokinetics	HIV-1-negative individuals n = 37	[63]
NCT02840474 (Gaudinski et al.)	2021	VRC01-LS /VRC07-523LS	Safety, pharmacokinetics, and antiviral activity	Viremic HIV-1-infected individuals, n = 16	[64]
NCT03015181 (Gaudinski et al.)	2019	VRC07-523LS	Safety and pharmacokinetics	HIV-1 negative individuals, n = 26	[65]
NCT02018510 (Caskey et al.)	2015	3BNC117	Safety and pharmacokinetics	Asymptomatic, ART-naive HIV-1 infected individuals, n = 7	[66]
NCT02446847 (Scheid et al.)	2016	3BNC117	Safety and antiviral activity incl. delay in viral rebound	ART-treated HIV-1-infected individuals with 3BNC117 sensitivity undergoing ATI, n = 13	[67]
NCT02018510 (Schoofs et al.)	2016	3BNC117	Antiviral activity incl. neutralization efficacy	Viremic HIV-1-infected individuals with 3BNC117-sensitive strains (8/9), n = 9	[75]
NCT02588586 (Cohen et al.)	2018	3BNC117	Safety, pharmacokinetics, and antiviral activity	ART-treated HIV-1-infected individuals undergoing ATI, n = 15	[68]
NCT02511990 (Caskey et al.)	2017	10-1074	Safety, pharmacokinetics, and antiviral activity	Viremic HIV-1-infected individuals, n = 33	[69]
NCT02960581 (Stephenson et al.)	2021	PGT121	Safety, pharmacokinetics, and antiviral activity	Viremic and ART-naïve HIV-1-infected individuals, n = 48 ART-treated HIV-1-infected individuals, n = 12	[70]

**Table 3:** Clinical trials of combination therapy with ≥2 broadly neutralizing antibodies.

Clinical Trial Study	Year	bNAb	Study Objective	Study Population	Ref.
NCT02825797 (Bar-on et al.)	2018	3BNC117 + 10-1074	Safety, pharmacokinetics, and antiviral activity	Viremic HIV-1-infected individuals with antibody sensitivity and have been on/off ART, n = 7	[77]
NCT028 (Mendoza et al.)	2018	3BNC117 + 10-1074	Antiviral activity incl. neutralization efficacy and latent reservoir	ART-treated and virally suppressed HIV-1-infected indivi- duals with antibody sensitivity undergoing ATI, n = 11	[79]
NCT02824536 (Cohen et al.)	2019	3BNC117 + 10-1074	Safety and pharmacokinetics	HIV-1-negative individuals, n = 24	[71]
NCT02825797 (Niessl et al.)	2020	3BNC117 + 10-1074	Antiviral activity, including Gag-specific CD8+ and CD4+ T-cell responses	Aviremic HIV-1-infected individuals undergoing ATI, n = 9	[86]
NCT03526848 (Geabler et al.)	2022	3BNC117 + 10-1074 + ART	Antiviral activity incl. neutralization efficacy and reservoir size	HIV-1-infected individuals in the presence or absence of ART, n = 26	[80]
NCT03571204 (Sneller et al.)	2022	3BNC117 + 10-1074	Safety, pharmacokinetics, and antiviral activity including reservoir size	HIV-1-infected individuals, who initiated ART during early/acute infection, undergoing ATI (n = 14), ART-naïve HIV-1-infected individuals with viraemic control (n = 5), n = 19	[81]
PACTR201808919297 244 (Mahomed et al.)	2022	VRC07-523LS + PGT121	Safety and pharmacokinetics	HIV-1-negative women, n = 45	[83]
PACTR202003767867 253 (Mahomed et al.)	2023	VRC07-523LS + CAP256V2- LS	Safety, pharmacokinetics, and antiviral activity	HIV-1-negative women, n = 42	[84]
NCT03205917 (Julg et al.)	2022	VRC07-523LS + PGDM1400 + PGT121	Safety, pharmacokinetics, and antiviral activity	HIV-1-negative individuals (n = 24) and viremic, ART-naïve HIV-1-infected individuals (n = 5), n = 29	[78]
NCT03928821 (Sobieszczyk et al.)	2023	VRC07-523LS + PGDM1400 + PGT121 + 10-1074	Safety, pharmacokinetics, and antiviral activity	HIV-1-negative individuals who were randomly assigned to either dual- (n = 18) or triple-bNAb therapy (n = 9), n = 27	[82]
NCT03565315 (Awan et al.)	2024	VRC07-523LS + 10E8VLS	Safety and pharmacokinetics	HIV-1-negative individuals, n = 8	[85]

**Table 4:** Clinical trials with bNAb(s) and latency-reversing agents (LRA) or immunostimulatory agents.

Clinical Trial Study	Year	bNAb	Study Objective	Study Population	Ref.
NCT02850016 (Gruell et al.)	2022	3BNC117 + romidepsin	Safety and efficacy on plasma viremia and latent reservoir	ART-treated HIV-1-infected individuals, n = 20	[93]
NCT03803605 (Gay et al.)	2022	VRC07-523-LS + vorinostat + ART	Safety and effects on viral reservoir	ART-treated HIV-1-infected individuals, n = 8	[94]
NCT03041012 (Gunst et al.)	2022	3BNC117 + romidepsin + ART	Safety and efficacy on plasma viremia and latent reservoir	Early-diagnosed HIV-1 individuals initiating ART, n = 55	[95]
NCT03588715 (Tebas et al.) CROI 2023	2022	3BNC117 + 10-1074 + pegylated-interferon Alpha2b	Safety and effects on viral replication and rebound	ART-treated HIV-1-infected individuals undergoing ATI, n = 14	[97]
NCT03837756 (Gunst et al.)	2023	3BNC117 + 10-1074 + lefitolimod	Safety, tolerability, and time to loss of virological control after ATI	HIV-1-infected individuals on ART undergoing ATI at week 3, n = 43	[96]

**Table 5:** Elimination half-lives of bNAbs, including longer-acting variants (-LS).

Broadly Neutralizing Antibody	Elimination T_1/2_ (Days)	Reference
VRC01	17	[66]
VRC01-LS	71	[63]
VRC07-523LS	29–66	[65,82,83,84]
3BNC117	16	[71]
3BNC117-LS	62 *	[101]
10-1074	23–27	[71,82]
10-1074-LS	80 *	[101]
PGT121	20–32	[82,83]
PGDM1400	20–25 11 *	[78,82] [78]
10E8VLS	8	[85]
N6LS	>30	[55]
CAP256V2LS	46	[84]

* in HIV-1-positive individuals.
